# Are isokinetic leg torques and kick velocity reliable predictors of competitive level in taekwondo athletes?

**DOI:** 10.1371/journal.pone.0235582

**Published:** 2021-06-09

**Authors:** Pedro Vieira Sarmet Moreira, Coral Falco, Luciano Luporini Menegaldo, Márcio Fagundes Goethel, Leandro Vinhas de Paula, Mauro Gonçalves

**Affiliations:** 1 Biomedical Engineering Program, COPPE, Federal University of Rio de Janeiro, Rio de Janeiro-RJ, Brazil; 2 Laboratory of Biomechanics, São Paulo State University (UNESP), Rio Claro-SP, Brazil; 3 Tech4Fight Sports Technology, Rio de Janeiro-RJ, Brazil; 4 Department of Sport, Food and Natural Sciences, Western Norway University of Applied Sciences, Bergen, Norway; 5 Laboratory of Biomechanics, Federal University of Minas Gerais, Belo Horizonte-MG, Brazil; Universidade Federal do Rio Grande do Sul, BRAZIL

## Abstract

The aim of this study is to analyze how isokinetic knee and hip peak torques and roundhouse kick velocities are related to expertise level (elite vs. sub-elite) in taekwondo athletes. Seven elite and seven sub-elite athletes were tested for kick-specific variables (KSV, composed of kinematic variables and power of impact) and for concentric isokinetic peak torque (PT) at 60°/s and 240°/s. First, KSVs and PTs were compared between groups, then PTs were correlated with KSVs. Parametric variables with larger effect sizes (Cohen’s *d*) were entered in a stepwise linear discriminant analysis (LDA), generating an equation to estimate competitive level. Between-group differences were found in hip flexors (p = 0.04, *d* = 0.92) and extensors (p = 0.04, *d* = 0.96) with PT at 240°/s. Hip flexion PT at 60°/s and 240°/s correlated negatively with kick time (R = –0.46, p = 0.0499 and R = –0.62, p = 0.01 respectively). Hip flexion torque at 60°/s correlated positively (R = 0.52, p = 0.03) with peak linear velocity of the foot (LVF) and power of impact (R = 0.51, p = 0.03). Peak torque of hip extension at 60°/s and hip abduction at 240°/s also correlated with LVF (R = 0.56, p = 0.02 and R = 0.46, p = 0.0499). Hip extension at 60°/s correlated positively with peak linear velocity of the knee (R = 0.48, p = 0.04). The LDA showed an accuracy of 85.7% (p = 0.003) in predicting expertise level based on hip flexion and extension torques at 240°/s and on knee extension velocity during the kick. The study demonstrates that hip muscle strength is probably the dominant muscular factor for determining kick performance. Knee angular velocity combined with hip torques is the best discriminator for competitive level in taekwondo athletes.

## Introduction

The most popular technique in taekwondo (TKD) combats is the roundhouse kick, or *bandal chagi* [1–3]. Defined as a multiplanar and multi-joint action [2–6], it is described as a *proximo-distal sequence*, in which structures nearest the center of the body (proximal segments) develop first in temporal order of joint movement, while distal segments lag behind, followed by relative acceleration of distal segments while proximal segments decelerate [7–9]. According to this principle, the highest possible velocity of the proximal segment, linked by interaction with the distal segments, plays an important part in determining the terminal velocity or impact magnitude (power, force, and kinetic energy) [3–5, 7, 9–13]. Angular acceleration of a segment is generated by muscle torques that control the proximal joint [6, 7, 14] and by the angular momentum transmitted to the next (more distal) segment [7, 14–16]. The resultant torque produced during the kick depends on the athlete’s coordinative capacity to maximize the agonist and minimize the antagonist torque [7, 16, 17].

*Bandal chagi* has two important phases, the *preparation phase* and the *kicking phase* [18]. The preparation phase is when significant force is exerted against the ground [19] and the kicking phase is when the foot travels along its aerial path toward the target [18]. The preparation phase (or stance phase) is considered as a slow but important phase of the kick, where high moments [19] and ground reaction forces [3] are produced by the hip and leg muscles [20, 21]. Due to the high acquired velocity of the segments and joints of the kicking leg [3, 20, 21], the kicking phase (or aerial phase) is the fast part of the roundhouse kick. Interestingly, the leg segment velocity acquired during the kicking phase is partly explained by the force and torques produced by the lower part of the body during the preparation phase [3, 19, 22]. Thus, as the speed is in part determined by the joint torques produced during both the fast [7, 9] and slow [19] phases of the kick, it is of scientific interest to study the association between kick performance and maximal capacity to produce torque in both slow and fast contraction.

The most popular way of evaluating torque in predetermined velocities is by isokinetic evaluation [23], because this kind of evaluation shows high reliability [22, 24], validity [17, 25–28], and control of speed and range of motion [23, 24]. According to Bell and Wenger [23], slow isokinetic velocity is defined as 1.75 rad/sec (≤ 100°/s) and fast as 3.51 to 5.24 rad/sec (201° to 300°/s). Additionally, different kinds of training (aerobic, force, plyometric, power, etc.), interacting with different genetic profiles (responsive to slow or fast training), define the kind of force predominantly developed in an athlete [29–31]. Bell and Wenger [23], in their review of physiological adaptations to isokinetic evaluation, observed that with an isokinetic evaluation velocity of 240°/s it is possible to detect a specificity effect of fast training (high-velocity resistance training), that is, the torque improvement that occurs only with an evaluation velocity of 240°/s (or at very similar velocities) but that does not occur at slow evaluation velocities (30°/s, 60°/s, 90°/s, or 96°/s). They observed that when individuals trained with lower contraction velocities, lower torque improvement was detected for 240°/s. In general, the same did not occur when the “fast” evaluation velocity was lower than 240°/s. Thus, 240°/s appears to be the minimum velocity for separating different kinds of neuromuscular training adaptations. Compared to other velocities (210°/s, 270°/s, 300°/s) considered as “fast,” 240°/s was precisely the velocity at which most of the fast force training effect was detectable [23]. On the other hand, isokinetic evaluation at 60°/s has proven to be sensitive for detecting force adaptations in some kinds of training with taekwondo athletes, such as those based on running, circuit training, and localized muscle endurance training [32] or plyometrics [33]. Thus, evaluating athletes at these two velocities appears to be a useful way to ascertain whether athletes of different levels have different force-velocity profiles.

Various studies have shown that martial arts athletes are capable of producing higher isokinetic peak torques than control groups or lower level athletes [27, 28] at different contraction speeds. Previous researchers [6, 27] have demonstrated that elite combat sports athletes show improved capacity to produce torque, compared with sub-elite athletes or non-athletes, at isokinetic velocities ranging from 30°/s to 400°/s. Fong and Tsang [26] consider isokinetic contraction velocities of 60°/s as low and 240°/s as high. Some authors have shown that the number of TKD training hours per week correlates positively with peak knee extensor (R = 0.639) and flexor (R = 0.472) torque at 240°/s, but not at 60°/s, nor with the ankle plantar flexors at any contraction speed. Pieter et al. [28] show that TKD practitioners reach higher hamstring isokinetic torques than non-athletes, at various contraction velocities. Elite TKD athletes have better performance than sub-elite athletes, kicking faster and more strongly and showing higher angular and linear velocities and lower kick times [5, 20, 34]. However, the isokinetic torque of TKD athletes has not yet been associated with the specific performance of a roundhouse kick.

Moreover, it can be difficult to know whether an athlete’s physical and technical performance corresponds to the biomechanical performance of an athlete of elite or sub-elite level. In this respect, a linear discriminant analysis (LDA) of specific biomechanical parameters can help us to rank and characterize them. Discriminant function analysis is a predictive model of membership of a group according to the athlete’s level and can be useful for ranking athletes based on the selected parameters [28, 35].

The objective of this study is therefore to analyze whether isokinetic leg torques and kick velocity are reliable predictors of competitive ranking in taekwondo athletes. Another important aim is to evaluate whether the capacity of athletes to produce muscle force (measured through isokinetic moments) in a very simple task (in terms of coordination: monoarticular, with the trajectory and velocity controlled by a machine) is associated with the speed of the *bandal chagi* kick (a complex task in terms of motor control). Three hypotheses will be addressed:

1. Elite athletes show higher isokinetic torques than sub-elite athletes, regardless of contraction speed.
2. Isokinetic torques correlate positively with peak linear and angular velocities and impact magnitudes obtained during the kick, and negatively with the temporal parameters of the kick.
3. A discriminant equation based on the significant variables for differentiating the groups is a strong predictor of ranking performance.

## Material and methods

### Participants

Fourteen black-belt TKD athletes (recruited by convenience sampling) participated in the study. They were divided into two groups: 7 elite athletes (five male and two female, finalists or semifinalists in national competitions; 23.6 ± 2.1 years; 69 ± 9.5 kg; 168 ± 5 cm; MD: 9.25, IQR: 7.92 years of training; 15.7 ± 4.7 hours per week of training) and 7 sub-elite athletes (five male and two female, medal-winners in competitions at state level); 22.4 ± 1.3 years; 66.8 ± 14.2 kg; 174 ± 11 cm; MD: 9.75, IQR: 8.50 years of training; 11.4 ± 5.1 hours per week of training). There were no significant differences between the groups for any of these variables (p > 0.1). The study was approved by the local ethic committee (CEP) of the Institute of Bioscience of the State University of São Paulo (UNESP—IB) with the number 058/2013, and all participants signed an informed consent form the same morning, before to start the data collection.

### Experimental design

In a cross-sectional design, participants were first evaluated through kinematic analysis and impact measurement during execution of the *bandal chagi* kick. After 10 minutes of passive rest, knee and hip isokinetic concentric torque curves were measured at two different velocities (60°/s and 240°/s).

### Data collection

Kinematics were measured using seven Vicon® MX13 cameras, sampled at 250 Hz. Thirty-nine marker reflectors were placed on each athlete, according to the Vicon® Plugin Gait Full Body (UPA and FRM) marker set [36]. After 15 minutes of warm-up, 9 *bandal chagi* kicks were performed, directed at a dummy (BoomBoxe®; see S1 video: **https://doi.org/10.6084/m9.figshare.9698741.v2**) equipped with a Daedo® TK-Strike 4.2 trunk protector, used for official competitions. The trunk protector registered the impact power in units of measurement appropriate for World Taekwondo Federation (WT) but not defined by the International System of Units (SI). An AMTI® OR6-6-2000 force plate, sampled at 2000 Hz, placed on the dominant rear kicking leg, determined the onset of the kicking phase [3]. The dominant leg was considered the “preferential member of attack” [37]. Ground reaction force (GRF) data were synchronized with the kinematic data using the Vicon® Nexus 2.0 program, having passed through a Vicon® GigaNet data synchronization box, which received digital data from the force plate’s data acquisition (DAQ) system (AMTI® model OPT-SC) and sent them to the computer analog board via a digital plugin (USB).

The isokinetic protocol consisted of four familiarization contractions, three submaximal and one maximal, followed by 15 seconds of rest, and five maximal concentric/concentric contractions, alternating between reciprocal knee flexion/extension, hip flexion/extension, and hip adduction/abduction movements [38], which are important movements for the kick [15], at speeds of 60°/s and 240°/s [26]. There were two-minute rest intervals between the pairs of reciprocal movements and during the alternation of contraction speeds [37]. The order of velocities and pairs of movements was randomized. Participants were instructed to make the contractions “as fast and strong as possible” [39] and there was verbal encouragement during the contractions. Analog torque, angle, and velocity curves were collected, using a Biodex® System 4 PRO isokinetic dynamometer, and sent to an analog input system (AIS) with BNC inputs (Noraxon® model 222 BNC), connected to the Noraxon® Telemyo DTS receiver. The AIS digitalized the signal at 3000 Hz and the DTS receiver sent the signals to a computer installed with the Noraxon® MR program, version 3.2. This program exported the signals to a.txt file and each file was then post-processed using a Mathworks® MATLAB routine.

Participants were seated, with the backrest fixed to the chair with straps and adjusted to 110° from the horizontal surface, i.e., with ~70° of hip flexion. The dynamometer axis was aligned with the greater trochanter of the femur. The knee started at 90° flexion, extended 90°, and returned to the starting position. Normally, peak knee flexion attained during *bandal chagi* kicks is ~90°, varying from about 90° to 100° [15, 40]. During the hip evaluation in the sagittal plane, the thigh started from 5° of flexion (0° = full extension) and covered 50° of flexion/extension [41], reaching 55° of peak flexion, an angle similar to the upper limit (mean + between-athletes standard deviation) of the peak angle obtained during *bandal chagi* by Kim et al. [15]. For the hip abduction/adduction evaluation, participants were placed in the lateral decubitus position with the knee fully extended; the rotational dynamometer axis was aligned with the ischial tuberosity and the dynamometer level was fixed to the dominant thigh. The range of hip abduction/adduction was between 0° and 45° [41, 42], with 0° defined as the legs in the horizontal position.

### Data processing

**Fig 1:**
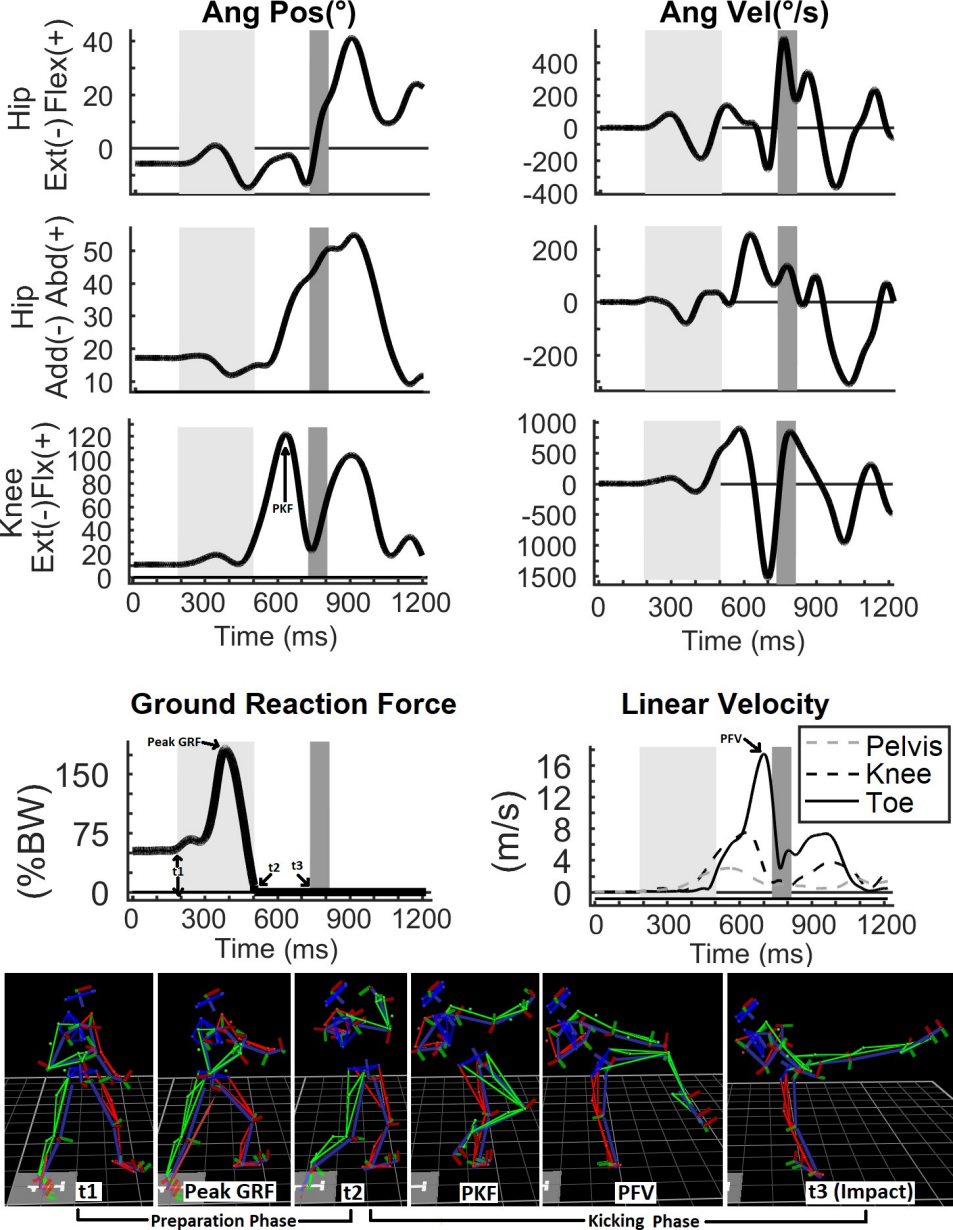
Angular position, velocity, and torque curves of a representative athlete performing a dynamometric evaluation of knee flexion and extension at 60°/s. The horizontal line is the velocity threshold separating the isokinetic phase from the other phases (acceleration and deceleration); the areas shaded in light green and gray represent the isokinetic extensor and flexor phases respectively.

Kinematic and ground reaction force (GRF) data were obtained with the Vicon® Nexus 2.0 program and exported to the Mathworks® MATLAB package. Rigid body segments, joint centers, and cardan angles were automatically calculated by the Nexus program, using the methods described in the *Plugin Gait Reference Guide* [36]. Through a MATLAB routine, data vectors of isokinetic angular position were numerically derived (in order of time) to obtain the angular velocity curves (Fig 1).

The torque and angle vectors obtained were then smoothed with a low-pass fourth-order Butterworth zero-lag filter (Bthw-0-lag) with a cut-off frequency of 20 Hz, corrected for segment weight according to Eq 1. The isokinetic velocity curve was filtered with a Bthw-0-lag with a cut-off frequency of 10 Hz, due to the high noise level compared to the angle data.

$$Final\;Torque_{\left(a\right)}=Initial\;Torque_{\left(a\right)}+Segment\;Weight\cdot Sin\;(\theta_{\left(a\right)})$$ (Eq 1)

where (a) represents the sample and θ the vertical orientation angle for the segment.

**Fig 2:**
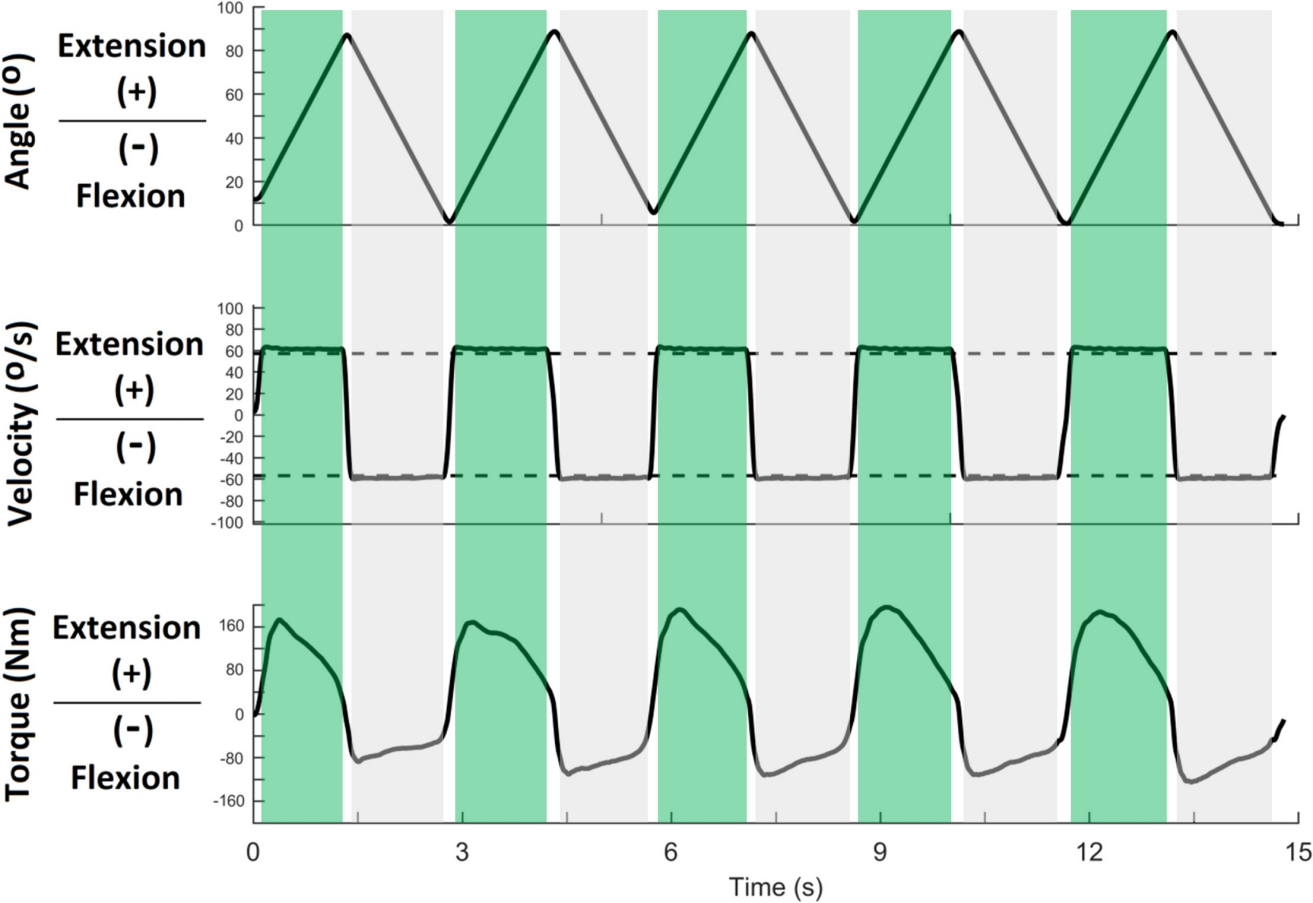
Curves resulting from the processing of kinematic and ground reaction force data and reconstruction of a kick by an elite athlete from the kinematic analysis. The subplots of the first column are the angular position curves, while the subplots of the second column show the respective angular velocities for hip and knee joints (1st to 3rd line of subplots). The 4th line of subplots contains the curves for the resultant ground reaction force and resultant linear velocities of pelvis, knee and foot. The light gray area represents the preparation phase and the dark gray area the impact phase; **t1**: onset of the preparation phase; **Peak GRF:** Peak ground reaction force; **t2:** end of the preparation phase and onset of the kicking phase; **PFK:** instant of peak knee flexion; **PFV:** instant of peak foot linear velocity; **t3:** end of the kicking phase and start of the impact.

Torque data were extracted from the isokinetic phase, considered as the phase in which the velocity reached at least 95% of the pre-defined velocity of 60°/s or 240°/s (Fig 2).

Kinematic and ground reaction forces were smoothed with a low-pass fourth-order Butterworth zero-lag filter, with cut-off frequencies of 10 Hz and 90 Hz respectively (defined using the residual analysis method).

Three kick events (onsets) were identified: *t*_*1*_ (onset of preparation phase), *t*_*2*_ (onset of kicking phase), and *t*_*3*_ (start of impact). The first, *t*_*1*_, is the instant corresponding to a systematic improvement (lasting at least 100 ms) in the resultant GRF above baseline (an average value of 50 ms from LED onset) amounting to 2.5% of the difference between peak GRF and the baseline value. After testing many window sizes (25 ms, 50 ms, 75 ms, 100 ms, 200 ms) and thresholds (1%, 2.5%, 5%) to determine GRF onset (*t*_*1*_), the best combination (with the fewest type I and type II errors) was 100 ms with 2.5% of the GRF difference. The second, *t*_*2*_, is the instant at which the GRF becomes zero. The third, *t*_*3*_, is when the foot touches the target, that is, when the dummy’s contact sensor overcomes a voltage threshold.

Next, the preparation time (PT = *t*_*2*_ – *t*_*1*_) and kicking time (KT = *t*_*3*_ – *t*_*2*_) were calculated (Fig 1). They correspond to the time during which the foot exerts pressure against the ground (PT) and the time during which it performs the aerial trajectory from the ground to the target (KT). During the KT, the maximal values obtained in the velocity curves for each linear and angular movement were used to determine the linear peak velocity (pelvis: anterior superior iliac spine; knee: lateral femoral condyle; foot: center of mass of the foot) and angular peak velocity (hip: flexion, extension, adduction, and abduction; knee: flexion and extension) of the anatomical points of the leg. The highest peaks of isokinetic torques, linear and angular velocities of the hip, knee, and ankle, and impact power were selected for further analysis.

### Statistical analysis

Normality of the data was verified by the Shapiro-Wilk test and homogeneity by Levene’s test. Analysis of Variance (ANOVA) and the Mann-Whitney test were used, for parametric and non-parametric statistics respectively, to compare isokinetic torque, impact magnitude, and selected kinematic kick speed data between groups. Pearson and Spearman correlation were used, for parametric and non-parametric statistics respectively, to analyze the relationship between peak isokinetic torques and performance kick data (kinematics and impact). To quantify the ANOVA effect size, Cohen’s *d* scores [43] were used according to Eq 2:

$$d=\frac{M_{A}-M_{B}}{\sigma }$$ (Eq 2)

where *M*_*A*_ and *M*_*B*_ are the two means and *σ* refers to the standard deviation for the population. Resulting *d* values of 0.2, 0.5 and 0.8 were considered to show a small, moderate, and large effect respectively. The equivalent to *d* for the Mann-Whitney test is *r*, according to Equ 3:

$$r=\frac{Z}{\sqrt{N}}$$ (Eq 3)

where *Z* is an output of the Mann-Whitney test and *N* is the sample size.

To describe the proportion of the total data variability in the ANOVA test accounted for by the effect under consideration, partial eta squared ($\eta_{p}^{2}$) was calculated according to Eq 4:

$$\eta_{p}^{2}=\frac{{SS}_{effect}}{{{SS}_{effect}+SS}_{error}}$$ (Eq 4)

where *SS_effect_* and *SS_error_* are respectively the sum of squares of the effect and of the error, outputs of the ANOVA test. For the Mann-Whitney test, eta squared (*η*^2^) was calculated according to Eq 5:

$$\eta^{2}=\frac{Z^{2}}{N}$$ (Eq 5)

The sample size calculation was based on preliminary data using 12 volunteers (6 elite and 6 sub-elite athletes). An average foot velocity difference of 1.8 m/s was observed between the groups of athletes. The a priori sample calculation showed that the ANOVA test would need to have 7 participants in each group for a minimum 80% chance of detecting a difference of 1.8 m/s in the linear velocity of the foot (critical F value = 4.75; actual power = 0.86). Considering the difficulty of recruiting more participants, the sample calculation for sensitivity revealed that with 14 athletes, the power of this study to detect a significant difference in torque between groups for *d* > 0.82 (critical F = 4.75) in the ANOVA test, and to detect significant correlations for coefficients of correlation greater than 0.58, would be more than 80%.

A linear discriminant analysis (LDA) was performed for the isokinetic torques and kinematic data to investigate how general muscle torque and specific kick performance can discriminate the expertise level: sub-elite = 1; elite = 2. Only data that showed linearity, normality, multicollinearity, homogeneity of variances, multivariate-normal distribution, and significant difference between groups through ANOVA were used in a stepwise discriminant analysis with the expertise level. Only two torque variables (hip flexion and hip extension, both at 240°/s) and two kinematic variables (AVKnExt: angular velocity of knee extension and LKV: linear knee velocity) met all the necessary assumptions to be manually imputed in the LDA. The selection criteria for the stepwise variable selection algorithm were set conservatively (default criteria of the SPSS software) to protect against type I error. The inclusion criteria considered variables with F values greater than 3.84 and the exclusion criteria applied to variables with F values lower than 2.71 after being combined in the generated function.

First, the LDA generated a general equation to classify the athletes based on the input variables. Then, the cross-validation was performed, based on Lachenbruch’s U method, that is, the successive classification of all cases but one to develop a discriminant function and then categorize the case that was left out. The process is repeated with each case left out in turn and produces a more reliable function. Lastly, a classification matrix was generated, containing the number of athletes correctly and incorrectly classified. The main performance classification metrics, based on the cross-validated classification matrix, were calculated according to whether athletes were correctly classified, with four alternatives: true elite (TE), false elite (FE), true sub-elite (TS) and false sub-elite (FS). Three main performance metrics were identified:

1. Sensitivity: the percentage of actual elite athletes correctly identified as elite, according to Eq 6:
Sensitivity=100*TE/(TE+FS) (Eq 6)
2. Specificity: the percentage of sub-elite athletes correctly identified as sub-elite, according to Eq 7:
Specificity=100*TS/(TS+FE) (Eq 7)
3. Accuracy: the percentage of participants correctly classified in the overall data, according to Eq 8:
Accuracy=100*(TE+TS)/(TE+TS+FE+FS) (Eq 8)

The main statistical tests were performed with the SPSS 18.0 program (SPSS Inc., Chicago, IL), taking *p* < 0.05 as the significance level. Effect sizes were calculated using MATLAB routines.

## Results

Table 1 shows comparative data between groups of kinematic and dynamic variables obtained during the kick and torques obtained during the isokinetic evaluations. The ANOVA indicated that in the elite group, the PT (F_(1,12)_ = 2.49, 0.01 < p < 0.05) and the KT (F_(1,12)_ = 2.93, 0.01 < p < 0.05) were borderline lower than in the sub-elite group. Peak angular velocity during knee flexion was significantly higher for the elite than for the sub-elite group (Mann Whitney: U = 11.0, p < 0.05). The ANOVA indicated that the linear peak velocity of the knee marker (F_(1,12)_ = 4.22, p < 0.05) and the angular velocity of knee extension (F_(1,12)_ = 6.18, p < 0.05) were higher in the elite than in the sub-elite group. Linear foot velocity was borderline higher in the elite group (F_(1,12)_ = 2.02, 0.01 < p < 0.05) than in the sub-elite group.

**Table 1:** Comparative data for kinematic and dynamic variables obtained during *bandal chagi* and isokinetic evaluations for each group of athletes.

Kind of variable	Kinematic variables	Athlete groups		Effect size		
		Elite	Sub-elite	(*d*)	($\eta_{p}^{2}$)	*P*
Time (ms)	PT	244±73	320±104	0.80	0.17	0.070
	KT	213±9	237±36	0.85	0.20	0.056
Peak Linear Velocities (m.s^-1^)	LFV	17.36±0.70	16.53±1.35	0.73	0.14	0.090
	**LKV**	8.20±0.44	7.73±0.41	**0.98**	0.26*	**0.031**
	LPV	3.14±0.34	3.03±0.28	0.35	0.03	0.265
Peak Angular Velocities (⁰/s)	**KnFlx**	994±242	832±322	**0.46**	0.21*	**0.049**
	**KnExt**	1584±78	1406±173	**1.12**	0.34*	**0.014**
	HipFlx	451±96	428±196	0.15	0.01	0.393
	HipExt	457±196	365±110	0.12	0.01	0.355
	HipAbd	441±127	365±101	0.65	0.11	0.121
Dynamic (AU)	Impact	44.57±1.33	44.66±2.20	0.14	0.02	0.320
Isokinetic Moment (Nm*Kg-1)	KnFlx 60⁰/s	1.60±0.18	1.60±0.24	<0.001	<0.001	0.500
	KnFlx 240⁰/s	1.23±0.19	1.22±0.12	0.02	<0.001	0.485
	KnExt 60⁰/s	2.79±0.30	2.61±0.43	0.15	0.02	0.310
	KnExt 240⁰/s	1.64±0.17	1.67±0.26	0.02	<0.001	0.500
	HipFlx 60⁰/s	2.22±0.31	2.02±0.32	0.64	0.11	0.123
	**HipFlx 240⁰/s**	1.78±0.17	1.59±0.21	**0.92**	0.23*	**0.042**
	HipExt 60⁰/s	2.65± 0.41	2.36± 0.31	0.77	0.16	0.079
	**HipExt 240⁰/s**	1.73±0.62	1.10 ± 0.56	**0.96**	0.25*	**0.035**
	HipAdd 60⁰/s	1.69±0.43	1.75± 0.41	0.15	0.01	0.393
	HipAdd 240⁰/s	0.92±0.23	0.86±0.48	0.16	0.01	0.390
	HipAbd 60⁰/s	1.93±0.36	1.83± 0.21	0.35	0.03	0.268
	HipAbd 240⁰/s	1.13±0.43	1.06±0.23	0.02	<0.001	0.500

**Key: PT:** Preparation time, the impulse phase of the kick; **KT:** Kicking time, the aerial phase of the kick; **LFV:** Linear foot velocity; **LKV:** Linear knee velocity; **LPV:** Linear pelvis velocity; **KnFlx:** Knee flexion; **KnExt**: Knee extension; **HipFlx:** Hip flexion; **HipExt:** Hip extension; **HipAdd:** Hip adduction; **HipAbd:** Hip abduction; **AU:** World Taekwondo Federation patented arbitrary unity of measurement (AU) of power impact. °/s: Isokinetic velocity in degrees per second; ***d*:** Effect size “Cohen’s *d*” from ANOVA test; **η2:** Percentage of explained variance of ranking classification in elite or sub-elite level

*: p < 0.05.

**Fig 3:**
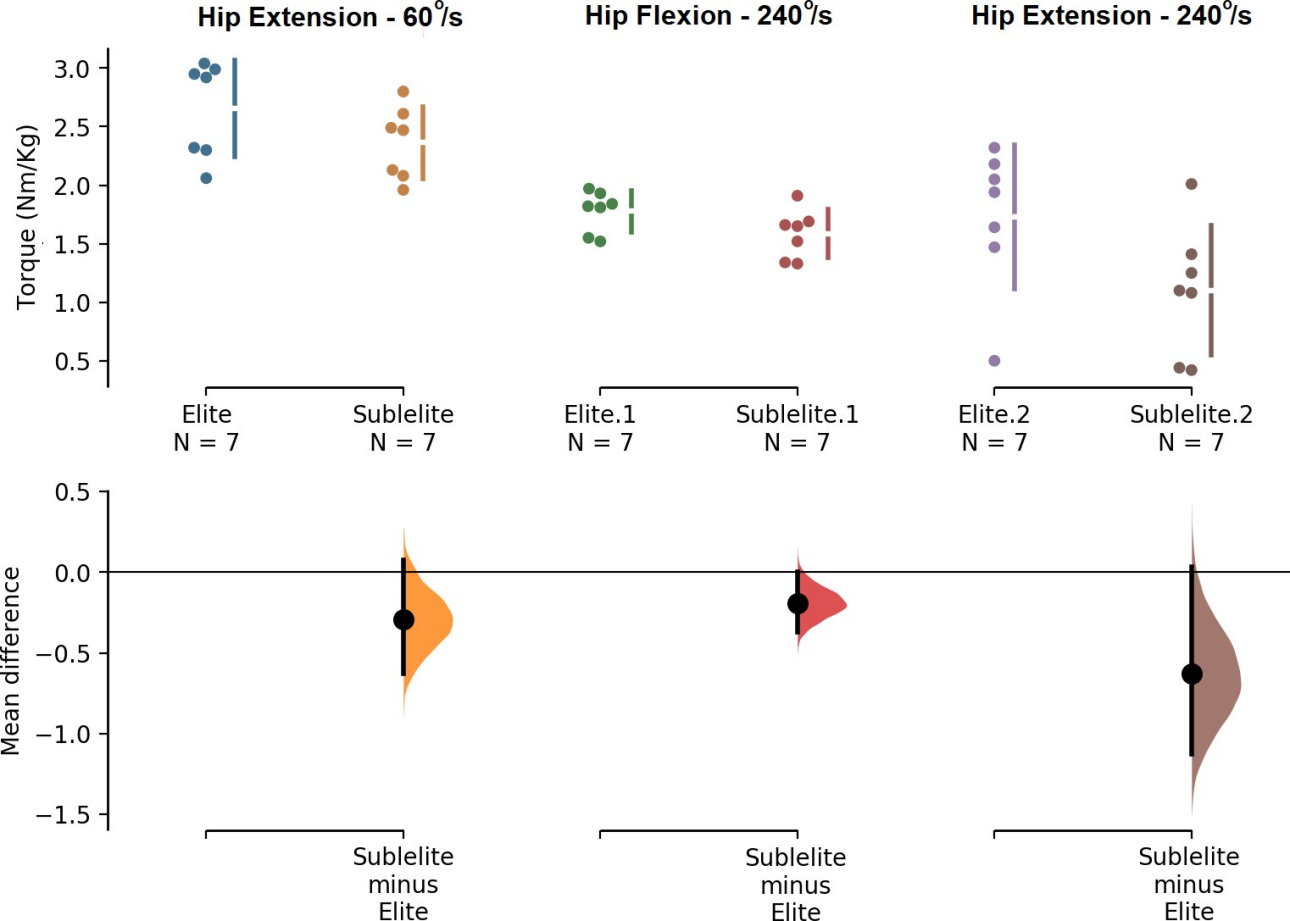
Comparisons for the most statistically significant torque variables. The mean difference for 3 comparisons are shown in the above Cumming estimation plot. The raw data is plotted on the upper axes; each mean difference is plotted on the lower axes as a bootstrap sampling distribution. Mean differences are depicted as dots; 95% confidence intervals are indicated by the ends of the vertical error bars [51].

Regarding isokinetic torques at 60°/s and 240°/s, for each joint, the results showed significant differences (Fig 3) between elite and sub-elite taekwondo athletes in peak torques for hip flexion (F_(1,12)_ = 3.56, p < 0.05) and extension (F_(1,12)_ = 3.953, p = 0.035) at 240°/s. The hip extensor torque at 60°/s was borderline higher in the elite group (F_(1,12)_ = 2.26, 0.01 < p < 0.05) than in the sub-elite group (Fig 3).

Except for the angular velocity of knee flexion (Cohen’s *d* = 0.46), all the significant differences were considered large (Cohen’s *d* > 0.80). Additionally, all the borderline differences obtained (0.73 ≤ Cohen’s *d* ≤ 0.85) were moderate to large.

Table 2 shows the correlations between the isokinetic peak torque and the roundhouse kick parameters.

**Table 2:** Coefficients of correlation between the isokinetic peak torques and the temporal, kinematic, and impact data obtained during the *bandal chagi* kick (n = 14 athletes as a single group).

Isokinetic Parameters			Kinematic Parameters during the Kick										Dynamic
			Time		Linear Velocities			Angular Velocities					Parameter
Movement		Velocity	PT	KT	LVF	LVK	LVP	KnFlx	KnExt	HipFlx	HipExt	HipAbd	Impact
KNEE	Flexion	60^o^/s	-.42 (0.07)	-.43 (0.06)	-.10 (0.37)	-.24 (0.20)	.41 (0.07)	.38 (0.09)	-.09 (0.38)	.21 (0.23)	-.13 (0.32)	.11 (0.36)	-.07 (0.40)
		240^o^/s	-.03 (0.46)	-.10 (0.37)	.17 (0.28)	-.20 (0.25)	.38 (0.09)	.38 (0.09)	.11 (0.35)	.11 (0.36)	-.27 (0.17)	.08 (0.39)	-.26 (0.18)
	Extension	60^o^/s	-.25 (0.20)	-.17 (0.28)	.35 (0.11)	.35 (0.11)	.35 (0.11)	.10 (0.37)	.22 (0.23)	.10 (0.36)	.27 (0.18)	.46* **(0.05)**	.20 (0.25)
		240^o^/s	-.34 (0.12)	-.03 (0.46)	.13 (0.33)	-.09 (0.38)	.44 (0.06)	-.06 (0.42)	-.25 (0.19)	.24 (0.20)	-.35 (0.11)	.08 (0.40)	.07 (0.41)
HIP	Flexion	60^o^/s	-.07 (0.41)	-.46* **(0.05)**	.52* **(0.03)**	.24 (0.21)	-.13 (0.33)	.16 (0.30)	-.24 (0.20)	.002 (0.50)	-.27 (0.18)	-.06 (0.42)	.51* **(0.03)**
		240^o^/s	-.36 (0.10)	-.62** **(0.01)**	.27 (0.17)	.25 (0.20)	.19 (0.25)	.44 (0.06)	-.09 (0.38)	.08 (0.39)	-.26 (0.19)	.20 (0.25)	.27 (0.17)
	Extension	60^o^/s	-.23 (0.22)	-.27 (0.18)	.56* **(0.02)**	.48* **(0.04)**	.25 (0.19)	.52* **(0.03)**	.24 (0.20)	.28 (0.17)	-.27 (0.18)	-.04 (0.44)	-.08 (0.39)
		240^o^/s	-.17 (0.28)	-.33 (0.12)	.31 (0.14)	.32 (0.13)	.02 (0.47)	.27 (0.17)	.27 (0.17)	.12 (0.34)	-.05 (0.43)	.16 (0.29)	-.08 (0.40)
	Abduction	60^o^/s	-.04 (0.44)	.26 (0.19)	.45 (0.06)	.36 (0.11)	-.20 (0.24)	-.17 (0.28)	.03 (0.47)	.12 (0.34)	-.04 (0.44)	-.09 (0.38)	.12 (0.35)
		240^o^/s	-.14 (0.32)	.24 (0.21)	.46* **(0.05)**	.25 (0.19)	.07 (0.40)	.03 (0.46)	-.11 (0.35)	.15 (0.30)	-.47 (0.09)	-.18 (0.27)	-.06 (0.41)
	Adduction	60^o^/s	.05 (0.43)	.40 (0.16)	-.01 (0.49)	.16 (0.29)	.04 (0.45)	.15 (0.30)	.07 (0.40)	.19 (0.26)	-.24 (0.21)	-.30 (0.15)	-.41 (0.14)
		240^o^/s	-.22 (0.23)	.06 (0.43)	.42 (0.07)	-.02 (0.47)	.30 (0.15)	.14 (0.32)	.04 (0.45)	.44 (0.06)	-.64* **(0.01)**	-.06 (0.42)	-.17 (0.28)

Correlations are presented as R (p value).

**Key: PT:** Preparation time, the impulse phase of the kick; **KT:** Kicking time, the aerial phase of the kick; **LFV:** Linear foot velocity; **LKV:** Linear knee velocity; **LPV:** Linear pelvis velocity; **KnFlx:** Knee flexion; **KnExt:** Knee extension; **HipFlx:** Hip flexion; **HipExt:** Hip extension; **HipAbd:** Hip abduction.

*: p < 0.05

**: p < 0.01.

Regarding the LDA analysis, the results (Tables 3 and 4) show that in the first analysis, hip flexion (TqHF_240_) and extension (TqHE_240_), at 240°/s predicted competitive level (multiple correlation with the discriminant equation: R = 0.61 and R = 0.58 respectively). The discriminant function revealed a significant association between the groups and all predictors, accounting for 47% of the between-group variability. The accuracy of the cross-validated classification showed that 64.3% of the participants were correctly classified.

**Table 3:** Results of the discriminant analysis of the discriminant functions (D_1,_ D_2_, and D_3)_.

					Centroid predictive value		Canonical correlation	
Discriminant equations					Elite	Sub-elite	R	p
**D**_**1**_ **=** TqHE_240_ * 1.454 + TqHF_240_* 4.337–9.352					0.872	–0.872	0.686	0.003
**D**_**2**_ **=** AVKnExt * 0.0074–11.13					0.665	–0.665	0.583	0.029
**D**_**3**_ **=** AVKnExt * 0.0074 + TqH_E240_ * 1.154 + TqHF_240_ * 5.44–21.8					1.542	–1.542	0.857	0.003
**Equations**	**TE (%)**	**FE (%)**	**TS (%) **	**FS (%)**		**Sensitivity (%)**	**Specificity (%)**	**Accuracy (%)**
**D**_**1**_	4 (57.1)	2 (28.6)	5 (71.4)	3 (42.9)		57.1	71.4	64.3
**D**_**2**_	6 (85.7)	2 (28.6)	5 (71.4)	1 (14.3)		85.7	71.4	78.6
**D**_**3**_	6 (85.7)	1 (14.3)	6 (85.7)	1 (14.3)		85.7	85.7	85.7

**Key: D**_**1**_: Discriminant equation based on isokinetic torque data; **D**_**2**_: Discriminant function based on kinematic data on kick performance; **D**_**3**_: Discriminant equation based on isokinetic torques and kinematic data on kick performance; **AVKnExt:** Peak angular velocity of knee extension; **LKV:** Linear knee velocity; **TqHE**_**240**_: Peak torque of hip extension at 240°/s; **TqHF**_**240**_: Peak torque of hip flexion at 240°/s; **Sensitivity:** the percentage of actual positives (elite) that are correctly identified as positive; **Specificity:** the percentage of failures (sub-elite) correctly identified as failures; **Accuracy:** the percentage of athletes correctly classified in their respective groups (elite or sub-elite); all the equations and metrics presented are based on the cross-validated classification matrix.

**Table 4:** Structure matrix: Pooled between-group correlations between discriminant variables and standardized canonical discriminant functions.

	Functions		
Variables	D_1_	D_2_	D_3_
TqHE _240_	0.609	--	0.344
TqHF _240_	0.578	--	0.327
AVKnExt	--	1.000	0.431
PKV	--	0.330^**a**^	--

**a:** Excluded from the model by the stepwise method; **D**_**1**_: Discriminant equation based on isokinetic torque data; **D**_**2**_: Discriminant function based on kinematic data on kick performance; **D**_**3**_: Discriminant equation based on isokinetic torques and kinematic data on kick performance; **TqHE**_**240**_: Peak torque of hip extension at 240°/s; **TqHF**_**240**_: Peak torque of hip flexion at 240°/s; **AVKnExt:** Peak angular velocity of knee extension; **LKV:** Linear Knee Velocity.

In the second analysis, the predictors were kinematic variables (AVKnEx and LVK). The discriminant function revealed a significant association between groups and only one significant predictor for the model (AVKnExt), accounting for 34% of the between-group variability. The cross-validated classification showed an accuracy of 78.6%.

Finally, a third analysis combined the peak torque and kinematically significant variables from the previous LDA. The discriminant function revealed a significant association between the groups and all the predictors, accounting for 74% of the between-group variability. The matrix structure revealed that all the variables were significant for the model, namely AVKnExt (R = 0.43), TqHE_240_ (R = 0.34), and TqHF_240_ (R = 0.33). The cross-validated classification showed an accuracy of 85.7%.

Table 4 contains the structure matrix of correlations between each explanatory variable of interest and the discriminant scores. Regardless of the equation (D_1_ or D_3_), TqHE_240_ showed higher correlations than TqHF_240_. For D_2_ and D_3_, AVKnExt was the variable that showed the highest correlation out of all the coefficients.

Table 5 contains the standardized canonical function coefficients for each significant variable for the three discriminant functions in the LDA analysis. These coefficients represent the relative weight of the selected variables in the generated score. Regardless of the equation (D_1_ or D_3_), TqHE_240_ showed the highest weight of all combined variables while TqHF_240_ showed the lowest weight for these functions.

**Table 5:** Standardized canonical function coefficients.

	Functions		
Variables	D_1_	D_2_	D_3_
TqHE _240_	0.854	--	1.041
TqHF _240_	0.830	--	0.678
AVKnExt	--	1.000	0.990

**D**_**1**_: Discriminant equation based on isokinetic torque data; **D**_**2**_: Discriminant function based on kinematic data on kick performance; **D**_**3**_: Discriminant equation based on isokinetic torques and kinematic data on kick performance; **TqHE**_**240**_: Peak torque of hip extension at 240°/s; **TqHF**_**240**_: Peak torque of hip flexion at 240°/s; **AVKnExt:** Peak angular velocity of knee extension.

## Discussion

### General results

Regarding the first hypothesis, elite and sub-elite athletes showed significant differences in their peak isokinetic torque values, in favor of the elite group. These differences were significant in the hip sagittal plane (flexion and extension) at high velocity (240°/s) and presented high effect sizes (Cohen’s *d* > 0.9). As the athletes did not differ in volume and frequency of training, but only in high velocity of contraction, it is believed that the difference may be due to at least one of two factors [30, 44]: 1) training quality and 2) conditional genetic factors for muscle power production. Tucker and Collins [44] state that “within the field of sports science, elite performance is understood to be the result of both training and genetic factors.” In this review, the authors describe the contributions made by deliberate practice and genetic factors to the attainment of a high level of sporting performance. They conclude that although deliberate training and other environmental factors are critical for elite performance, they cannot by themselves produce an elite athlete. Rather, individual performance thresholds are determined by an athlete’s genetic make-up, and training can be defined as “the process by which genetic potential is realised”. This means that elite sporting performance is the result of the interaction between genetic and training factors. Referring specifically to muscle power heritability (percentage genetic influence), they found that in the studies they reviewed, although figures ranging from 15% to over 90% are reported, all studies show that muscle mass and strength have a heritable component. Thus, Beunen et al. [30] find that heritabilities for dynamic strength of arm and leg muscle groups range from 29% to 87%. In our study, however, the differences between the groups of athletes manifested themselves only in specific muscle groups (hip flexor and extensor muscles); if they were the result of significant differences in genetic profile between the groups, one would expect that differences in ability to generate rapid strength would also be found in other muscle groups. We consider the first hypothesis, training quality, to be the more probable explanation. If this is the case, it suggests that the quality of strength training with high-speed sagittal hip movements should have a significant effect on the sport-specific classification.

When it comes to contraction speed, isokinetic torques of the hip joint, evaluated at both high and low velocity during the hip movement, correlated with essential parameters (namely KT, LFV, KnFlx, and Impact) of the kinematic performance of the kick. It is possible to explain the significant correlations of fast isokinetic contraction by the principle of training specificity, because martial athletes reach high angular velocity in their hip movement while performing the kick [6, 17, 45]. However, during the initial phases of the kick, the angular velocities are normally relatively low [6, 17, 45], and the acceleration of the lower limb segments occurs at this point, manifested at low contraction velocities during the start of muscle torque production [7, 19].

### Knee joint: Group comparison and correlations with kinematic variables

For the knee joint, the flexion and extension torques did not differentiate the two groups, and peak torque during knee flexion or extension did not correlate with kinematic variables, nor with impact. These results show that in expert athletes, the inter-individual differences in knee torque (see Table 1) did not affect the competitive results. Presumably, this happened because the athletes in the present study did not significantly differ in their weekly amount of training or in the total time spent training. Conversely, Fong and Tsang [26] found significant correlations between weekly specific taekwondo training and knee flexion and isokinetic extension torques, but only at high speed (240°/s). Our results do not support the findings of Sbriccoli et al. [6], who found that for knee flexion, elite karateka produced higher torques than amateurs in all evaluated velocities from 30°/s to 400°/s.

### Correlations between isokinetic moment and kicking time

In our study, peak isokinetic torque during hip flexion and kicking time correlated negatively at both contraction velocities. This is an important result, because shorter kicking time leaves the opponent less time to counterattack. For example, the specific reaction time in taekwondo varies from ~250 to ~350 ms and differs between groups according to expertise level [20]. Taking standard deviation into account, our kicking time (KT) values vary from 204 to 273 ms. Consequently, some of our participants kick more slowly (KT > 250 ms) than the reported reaction time of some TKD athletes. Naturally, this will affect kick efficacy. Therefore, strong, powerful hip flexors will generate more effective kicks. This finding is to be expected, because during the kicking phase, the dominant lower limb is free (off the ground) and unconstrained, reducing inertial resistance (inertial moment) against muscle action [46]. According to classical equations of mechanics (Eqs 9–11), this increases the ability of the hip flexors to produce velocity with a given muscle force. Eq 9 shows that for free rigid bodies (thigh + leg + foot), joint moment is the inertial moment multiplied by the angular acceleration (of the hip joint in this case). Therefore, for a given muscle force, velocity is inversely proportional to the inertial moment:

M=I∙α (Eq 9)

where *M* is the joint moment, *I* is the inertial moment, and *α* is the angular acceleration. From the muscle point of view,

M=F∙r∙cos(φ) (Eq 10)

where *M* is the joint moment, *F* is the muscle force, *r* is the distance from the joint center (the hip in this case) to the insertion of the tendon (of the hip flexor muscles in this case) on the bone (the femur in this case), and *φ* is the tendon insertion angle. Consequently, angular velocity (*ω*) is given by:

$$\omega =\int \alpha \;dt=\int M/I\;dt=\int \frac{F∙r∙cos(\varphi )}{I}dt$$ (Eq 11)

Additionally, during most of the kicking time, the hip flexes simultaneously with the knee, reducing the moment of inertia [46]. This appears to be a reasonable explanation for why the value of the coefficient of correlations between kicking time and hip isokinetic moment in the modulus was higher at 240°/s than at 60°/s. Analyzing Eqs 9 to 11, we can see that in the isokinetic mode, slow contraction speed simulates a sporting situation in which the inertial moment is high, while at higher velocities such as 240°/s it simulates a situation in which the inertial moment (*I*) is low; in other words, reducing the force in the isokinetic mechanical motor to enable the user to reach 240°/s has the same mathematical effect on hip angular velocity (*ω*) as reducing *I* during a sporting movement such as a kick. One might argue that during kicks, angular velocities are much faster than 240°/s; however, we found that for hip movements the average peak velocities during the kicks ranged from 365°/s to 457°/s. Additionally, muscle force modeling [34] has demonstrated that hip flexor muscles develop maximal force before the hip reaches its maximal angular velocities. It starts to develop force while the foot is still on the ground, but high levels of muscle force are maintained during almost all the aerial kicking phase [34]. However, in the present study, no significant correlations with preparation time were found.

### Correlations between isokinetic moments and kick angular velocities

Most of the expected correlations between joint torques and angular velocities were not significant, with two exceptions: 1) the correlation between hip extension at 60°/s and knee flexion angular velocity, and 2) the correlation between knee extension at 60°/s and hip abduction angular velocity. The first of these may perhaps be explained by the biarticular function of the hamstrings, flexing the knee and extending the hip [47]. During the roundhouse kick, biceps femoris activation occurs about 200 ms before significant production of muscle force, because of the electromechanical delay [20, 34]. For this reason, activation of the hamstrings and the start of the knee flexion action occur before the kicking phase, simultaneously with the hip extension movement of the preparation phase [20, 34]. One possible explanation for the second correlation is the biarticular muscular function of the tensor fasciae latae (TFL), a hip abductor and knee extensor [18, 20, 47]. The TFL is the first muscle to be activated in roundhouse kicks performed by elite athletes, whereas this is not the case with sub-elite athletes [20, 34]. It shows a waveform with 2 peaks, a lower peak during the preparation phase and a large one (70%– 100% of maximum voluntary contraction) in the kicking phase, synchronized with the rectus femoris, an important knee extensor [18, 20, 34]. It follows that an improved capacity to use this muscle during the kick may share a common factor of influence with the development of knee extensor torque.

An unexpected result was the negative correlation between peak torque during hip adduction at 240°/s and the angular velocity of hip extension during the kick. Hip adductor muscles reach the peak of activation during the aerial phase of the kick, when the knee is extending [18, 20]. Consequently, high forces (185–433 N) are generated in these muscles [21], which may act as synergists for hip flexion [47]. In this case, strong hip adductors will act to decelerate the hip extension angular velocity [18, 48].

### Correlations between isokinetic moments and linear velocities

Significant positive correlations were found between peak isokinetic torques of hip flexion (at 60°/s/), extension (at 60°/s), and abduction (at 240°/s) and the peak linear velocity of the foot during the kick. Sørensen et al. [7] have shown for the front kick that the hip flexors must counteract the hip extension reaction moment, caused by inertial torque of the knee during its angular acceleration toward extension. When the knee is extending, hip angular velocity is relatively low [7], allowing larger hip flexion muscle forces, according to Hill’s model [49]. The correlation between hip extensor torque at slow isokinetic speed and linear velocity of the foot can be explained by proximal-distal transmission of momentum [7, 22]. The action of hip extensor muscles, at the correct time, can potentiate proximal-distal momentum transmission, as demonstrated in our previous studies, where the peaks of linear foot velocity and knee extension angular velocity are preceded by an active hip flexion deceleration [20, 21].

The significant correlation found between hip abductor isokinetic torque and foot linear velocity was significant only at 240°/s. Hip abduction torque accelerates the thigh, and consequently the foot segment. Before impact, the knee extends and suddenly increases the moment of inertia of the entire leg relative to the hip. At this point, the hip abducts, and the increased moment of inertia tends to reduce the angular acceleration. Therefore, stronger abductors will probably provide more speed to the foot during the kick and explain the significant correlation.

Another interesting result was that hip extensor moment at 60°/s correlated positively with peak knee velocity. One possible explanation for this association is that a faster athlete produces higher hip extensor torque on the supporting leg than a slower athlete, transferring more kinetic energy from the supporting thigh to the pelvis and from the pelvis to the kicking thigh [19], impacting linear velocity to the knee, since it is the sum of the linear velocity of the proximal part of the pelvis (kicking hip) and the product of the angular hip flexion by the thigh length (Eq 12):

LKVi=LHVi+(ωH*LT)i, (Eq 12)

where LKV is linear knee velocity, LHV is linear hip velocity, ⍵H is the angular velocity of the kicking thigh and LT is the length of the thigh, while i is the specific axis of interest (x, y, or z).

### Correlation between moments and kick impact

The only torque that significantly correlated with impact magnitude was that produced during isokinetic hip flexion at 60°/s. With hip flexor torque at the slow isokinetic velocity as an independent variable, a positive correlation was also be expected, because during impact there is high resistance (from the dummy’s mass) against the movement, causing a sudden deceleration. Our results are in line with Vagner et al. [50], who found a significant multiple regression (R^2^ = 54%) between the two torque variables, hip flexor and hip extensor torque, at low velocity (90°/s) with kick impact (force) in front kicks, measured with a standard piezoelectric force plate. This shifts the force-velocity relationship to the left of Hill’s model [49]. It means that with high loads acting against the impact, the capacity to generate velocity decreases.

### Considerations about coordination

All the correlations obtained in this study had low to moderate effect sizes. The major part of kick performance in taekwondo may therefore be associated with other parameters, such as technique and coordination. Some studies confirm the importance of coordination for kick speed and impact intensity, by demonstrating that kick performance is influenced by factors such as intra-limb [16] and inter-joint [10] coordination, proximal-to-distal transmission of momentum (and kinetic energy) [7, 9, 22], muscle co-contraction [4, 6, 20, 34, 45], and using the stretch-shortening cycle [14, 22].

### Discriminant analysis

This study also identifies three significant discriminant equations (Table 3) for expertise level, based on a) peak torques during isokinetic muscle contraction, b) kinematic variables during kicking performance, and c) a combination of both. The last equation (D_3_) supports our third hypothesis, that a discriminant equation combining kinematic and torque variables would be a strong predictor of ranking performance. With this equation, based on hip flexion and extension torques at 240°/s and knee angular velocity, we found a high level of statistical performance for all (elite or sub-elite) group prediction parameters; specifically, accuracy (“hit-ratio”), sensitivity, and specificity attained a success rate of 85.7% for expertise level. The isokinetic torque variables represent the muscle force and power capacity required to generate torque in a simple (monoarticular) task, relatively independent of motor coordination and specific technical skills [6, 22]. The angular velocity of knee extension during a kick represents the opposite, that is, the capacity to use muscle force, influenced by technical skills [11, 14, 16, 22], to generate a sport-specific performance. Such a neuro-motor task should include the ability to take advantage of proximal-to-distal momentum transmission [7, 9, 22]. For example, in discriminant function D_2_, the angular velocity of the most distal analyzed joint (knee extension) discriminated elite from sub-elite athletes with the higher effect size obtained (*d* = 1.12) and it was a good predictor for categorizing the Brazilian taekwondo athletes in their respective groups; that is, those scoring above and below than 1504°/s can be categorized as elite or sub-elite, respectively.

It is interesting to note that if the standardized canonical function coefficients (Table 5) are used to understand which variables have high discriminant power in the LDA models, different results will be found compared to those obtained when the structure matrix correlations are used (Table 4). If we have to choose, we recommend opting for the structure matrix correlations, because when the knee angular velocity variable was analyzed separately to generate the discriminant equation D_2_, the accuracy of the model (Table 3) was greater than when the two hip torque variables were analyzed separately in D_1_. The opposite happened with the canonical correlation. This indicates that the standardized coefficients are related more to the canonical correlation and the structure matrix correlations to the model’s accuracy.

### Limitations of the study

As limitations of this study, we would point out that the kick impact data need to be interpreted with caution, since the sensors used had no validations of measurement errors and the unit of measurement is not part of the international system. Moreover, comparting athletes from different levels (i.e., international, national, state, and regional) are necessary to support the scoring reference values to better categorize the athletes, level. Additionally, future longitudinal studies are needed to confirm the effect of training designed to improve muscle torques of selected joints on the specific performance of TKD movements. A further limitation is that inverse dynamics was not used to calculate the torques and the momentum transmitted from proximal to distal segments during the kick, which could have enriched the discussion of the results.

## Conclusion

Isokinetic hip flexion and extension torques are specifically associated with TKD performance. Such torques are significant both in differentiating the competitive ranking of athletes and for their correlations with the kinematic performance and impact of kicks. Muscle force production capacity is important for taekwondo performance, not only at high contraction speed (240°/s), but also at low speed (60°/s). Slow-speed torques significantly correlated in our study with critical parameters of kick performance, including linear foot velocity, impact magnitude, and kick time. Additionally, the combination of isokinetic torque production (hip sagittal torque at 240°/s) with a sport-specific kinematic performance parameter (angular velocity of knee extension), using a linear discriminant function, yielded a strong predictor of the athletes’ expertise level.

Three main practical conclusions can also be drawn from this study:

- Experienced taekwondo athletes aiming to improve their competitive level and training efficiency should strengthen the muscles that control hip joint movements (flexion, extension, and abduction).
- Such strength training should be performed both at low loads with high speed and at high loads with relatively low speed. On this basis, we can speculate that training hip muscle strength with overload (elastic tubes, free weights, pulley exercise, etc.) has greater potential to improve kick impact than training other muscle groups.
- The discriminant analysis helps us to understand and calculate to what extent training is needed in order to attain the level of the best athletes within the reference range.

## Supporting information

S1 Video Demonstration of the data collection and rigid body diagram of an athlete performing a *bandal chagi* kick, followed by a kinematic analysis resulting from a number of *bandal chagi* kicks performed by another athlete included in the study. Figshare, uploaded on September 4, 2019. Available at: https://doi.org/10.6084/m9.figshare.9698741.v2.
(TXT)

S1 Data Data used for statistical analysis in an Excel spreadsheet. **Key: PIT:** Peak isokinetic torque; **AVK:** Angular velocity during the kick movement; **PT:** Preparation time, the impulse phase of the kick; **KT:** Kicking time, the aerial phase of the kick; **LFV:** Linear foot velocity; **LKV:** Linear knee velocity; **LPV:** Linear pelvis velocity; **KnFlx:** Knee flexion; **KnExt:** Knee extension; **HipFlx:** Hip flexion; **HipExt:** Hip extension; **HipAdd:** Hip adduction; **HipAbd:** Hip abduction; **Impact:** Impact power value, in units of measurement patented by World Taekwondo. **60:** 60 degrees per second in isokinetic evaluation; **240:** 240 degrees per second in isokinetic evaluation.
(XLSX)
